# Moderating Effects of Muscle Fitness on the Associations Between Work Stress, Burnout, and Well-Being Among White-Collar Workers

**DOI:** 10.3390/healthcare14040468

**Published:** 2026-02-12

**Authors:** Shu-Ling Huang, Wei-Hsun Wang, Ren-Hau Li, Hsuan-Yu Chen, Feng-Cheng Tang

**Affiliations:** 1 Department of Psychology, Chung Shan Medical University, Taichung 402, Taiwan; shuling@csmu.edu.tw (S.-L.H.); davidrh@csmu.edu.tw (R.-H.L.); 2 Room of Clinical Psychology, Chung Shan Medical University Hospital, Taichung 402, Taiwan; 3 Department of Orthopedics, Changhua Christian Hospital, Changhua 500, Taiwan; 132072@cch.org.tw; 4 Department of Post-Baccalaureate Medicine, College of Medicine, National Chung Hsing University, Taichung 402, Taiwan; 5 Department of Golden-Ager Industry Management, Chaoyang University of Technology, Taichung 413, Taiwan; 6 Department of Clinical Psychology, Tsyr-Huey Mental Hospital, Kaohsiung 831, Taiwan; j842251022@gmail.com; 7 Department of Occupational Medicine, Changhua Christian Hospital, Changhua 500, Taiwan; 8 Graduate Institute of Clinical Medicine, College of Medicine, National Chung Hsing University, Taichung 402, Taiwan

**Keywords:** health promotion, muscle fitness, burnout, well-being, white-collar

## Abstract

Background/Objectives: White-collar workers experience a unique dual burden of high psychological demands and prolonged static loading, creating a need to understand how physical resilience may mitigate these stressors. This study investigated the moderating role of specific muscle fitness components in the associations between work stress, burnout, and well-being among white-collar workers. To address the gap in task-specific physical resilience, we employed a cross-sectional design involving 321 full-time employees. Methods: Work stress (job control and demands), burnout, and well-being were assessed via structured questionnaires, while grip strength, abdominal endurance, and back muscle endurance were objectively measured. Results: Results indicated that the muscle fitness components were not directly associated with either burnout or well-being. However, the moderation model for burnout was significant (*F* = 15.837, *p* < 0.001; adjusted *R*^2^ = 0.278), where back muscle endurance significantly moderated the association between psychological job demands and burnout (β = −0.121, *p* < 0.05), whereas no such moderating effect was observed for well-being. In contrast, no such moderating effect was observed for well-being, nor did grip strength or abdominal endurance exhibit significant buffering effects on either psychological outcome. Conclusions: These findings demonstrate the relevance of task-specific physical resources in sedentary environments, specifically that back endurance functions as a buffer against burnout but may be insufficient to directly enhance overall well-being. The results suggest that while integrating task-specific physical assessments is vital for burnout prevention, psychosocial organizational support remains essential for fostering comprehensive well-being.

## 1. Introduction

Work-related stress has emerged as a significant public health challenge, particularly among white-collar workers who are frequently exposed to high psychological demands, intensive time pressure, and prolonged sedentary behavior [1,2]. Sustained exposure to such stressors is consistently linked to adverse psychological outcomes, most notably burnout and diminished well-being [3,4]. Burnout is conceptualized as a multidimensional psychological syndrome resulting from chronic occupational stress, characterized by emotional exhaustion, depersonalization, and a reduced sense of personal accomplishment [5]. Conversely, well-being is a broader positive construct encompassing optimal psychological functioning and life satisfaction [6,7,8,9]. Together, these constructs provide a comprehensive assessment of both the negative and positive dimensions of occupational mental health.

Recent research within the positive occupational psychology framework emphasizes that the absence of ill health (e.g., burnout) does not equate to flourishing or well-being. This “dual-continuum model” proposes that mental health ranges from languishing to flourishing [10], and that the resources required to promote well-being often differ from those that mitigate exhaustion [11]. In the Taiwanese context, deteriorating psychosocial work conditions—such as increasing job demands and the growing prevalence of nonstandard work arrangements—have been documented over time, highlighting the salience of psychosocial stress in Taiwanese workplaces [12]. While muscle fitness has been shown to help mitigate the physical strain that may precede burnout, its role in fostering positive cognitive–affective states remains a subject of ongoing debate [13,14]. Understanding why physical resources may buffer strain without necessarily enhancing flourishing is therefore theoretically important for the development of more targeted interventions.

Beyond its general health benefits [15,16,17], physical fitness facilitates adaptive responses to psychological stress [18,19]. While research has traditionally focused on cardiorespiratory fitness (CRF) [20], muscle fitness—specifically grip strength and trunk endurance—remains underexamined [21,22,23]. For white-collar workers, trunk and abdominal endurance are vital for maintaining postural stability during the prolonged static loading characteristic of modern office work [24,25]. These physical capacities may influence workers’ tolerance for sustained demands, suggesting a plausible pathway through which muscle fitness interacts with psychological stress.

White-collar environments involve prolonged sedentary behavior and static postures, which impose significant biomechanical strain on the spinal structures [26]. Insufficient endurance in the trunk and back muscles is a known risk factor for postural instability and musculoskeletal discomfort [27,28]. Over time, these physical stressors can induce chronic fatigue, depleting the psychological resources necessary to cope with work demands and potentially accelerating the development of burnout [29,30]. In contrast, superior back muscle endurance may enhance spinal stability and attenuate the physical discomfort associated with high job demands, thereby protecting against the cumulative effects of stress [24].

Finally, the Job Demand-Control (JDC) model posits that high-strain conditions increase vulnerability to stress-related disorders [31]. Although job control can mitigate burnout [4,32], variability in individual responses suggests that individual-level resources, such as physical fitness, may moderate these effects [33,34]. Moving toward “precision prevention,” the present study aims to examine the moderating role of multiple muscle fitness components—including grip strength, abdominal endurance, and back muscle endurance—in the associations between work stress, burnout, and well-being among white-collar workers. By integrating psychosocial job characteristics with objective physical assessments, this study seeks to clarify whether specific muscle fitness indices function as critical stress-buffering resources in contemporary office-based settings.

## 2. Materials and Methods

### 2.1. Study Design and Participants

A cross-sectional study design was employed, with all procedures conducted in accordance with the Declaration of Helsinki. Ethical approval was granted by the Institutional Review Board of Chung-Shan Medical University Hospital, Taiwan (CSHIRB No: CS18263). Sample size requirements were calculated using G*power 3.1.9.7 software, based on an effect size of *f*^2^ = 0.10, alpha error probability = 0.05, power = 0.90, and ten predictors, indicating that at least 215 participants were required. The target population comprised 1196 full-time white-collar employees at a glass product manufacturing facility. Participants were selected from the human resources registry using a systematic random sampling approach. To ensure objective selection, every third individual (*k* = 3) was invited based on employee ID numbers, such that one individual was selected for every three eligible workers.

Prior to data collection, all potential participants were provided with detailed information regarding the study objectives, and the voluntary nature of participation was emphasized. Written informed consent was obtained from all participants, and confidentiality was strictly maintained. For safety purposes, an occupational physician conducted pre-test screenings and provided on-site supervision throughout the assessments. Testing was terminated immediately if participants reported discomfort or if the physician observed signs of excessive strain, following standardized interruption protocols. Anthropometric measurements and fitness assessments were performed by trained research assistants following standardized protocols. Of the 380 workers initially invited, complete data were obtained from 321 participants, yielding a valid response rate of 84.5%.

### 2.2. Data Collection Procedures

Data were collected through a combination of self-administered questionnaires, anthropometric assessments, and standardized fitness tests. The questionnaire gathered data on demographic characteristics, work-related factors, burnout, and well-being. Demographic variables included age, gender, marital status, educational level, and perceived economic status. Work-related variables encompassed shift work status, average weekly working hours, job control, and psychological job demands.

#### 2.2.1. Assessment of Work Stress

Psychosocial work stress was assessed using the Chinese version of the Job Content Questionnaire (C-JCQ) [35], validated for Taiwanese populations. The C-JCQ is derived from the original instrument proposed by Karasek et al. [36] and includes 14 items across two subscales: job control (9 items, covering skill discretion and decision authority) and psychological job demands (5 items). Responses were recorded on a four-point Likert scale (1 = strongly disagree to 4 = strongly agree). Weighted scores were calculated following the procedures described by Cheng et al. [30], with higher scores indicating higher job control or demands. In this study, Cronbach’s alpha coefficients were 0.685 for job control and 0.763 for psychological job demands.

#### 2.2.2. Assessment of Burnout

Burnout was measured using the validated Chinese version of the Copenhagen Burnout Inventory (C-CBI) [37] based on the original scale by Kristensen et al. [38]. The C-CBI consists of 13 items assessing personal burnout (6 items) and work-related burnout (7 items). Participants responded on a five-point Likert scale ranging from never to always. Following established protocols, responses were transformed to a 0–100 scale, with higher scores reflecting greater burnout severity. An overall burnout score was calculated by averaging the two subscales. In the current sample, the overall burnout scale demonstrated excellent internal consistency (Cronbach’s α = 0.938).

#### 2.2.3. Assessment of Well-Being

Subjective well-being was assessed using the five-item World Health Organization Well-Being Index (WHO-5) [8]. The WHO-5 measures cheerful mood, calmness, vigor, restfulness, and interest in daily life over the past two weeks. Responses were rated on a six-point Likert scale (0 = at no time to 5 = all of the time). Total scores were summed, with higher scores indicating better well-being. The WHO-5 is widely recognized for its reliability and validity [9,39]. In the present study, the scale exhibited excellent internal consistency (Cronbach’s α = 0.940).

#### 2.2.4. Body Mass Index (BMI)

Height and weight were measured using standardized equipment, and BMI was calculated as weight (kg)/height^2^ (m^2^). BMI categories were defined according to the Health Promotion Administration (HPA) of Taiwan: underweight (BMI < 18.5), normal weight (18.5 ≤ BMI < 24.0), overweight (24.0 ≤ BMI < 27.0), and obese (BMI ≥ 27.0) [40].

#### 2.2.5. Muscle Fitness Measurements

Muscle fitness was evaluated using three indicators: grip strength, abdominal muscle endurance, and back muscle endurance. Grip strength was measured using a microFET® digital HandGRIP dynamometer (Hoggan Scientific, Salt Lake City, UT, USA) to the nearest 0.1 kg; the higher value of two trials was used. Abdominal and back muscle endurance were assessed by the maximum number of sit-ups and trunk extensions completed within 60 s, respectively. Performance values were classified into five levels (very poor to excellent) based on age- and sex-specific reference standards for Taiwanese workers [41]. To ensure comparability across different demographic groups, these standardized levels were treated as continuous variables in the subsequent statistical analyses.

### 2.3. Statistical Analysis

Descriptive statistics summarized participant characteristics. Independent-sample t-tests and one-way ANOVA examined differences in burnout and well-being across groups. Pearson’s correlation coefficients assessed associations between continuous variables. Multiple linear regression analyses evaluated the moderating effects of muscle fitness on the relationship between work stress and psychological outcomes. To ensure a parsimonious model, only covariates showing significant bivariate associations with burnout or well-being were controlled. Three models were constructed for each outcome: Model 1 examined grip strength and its interactions with job control and demands; Model 2 examined abdominal endurance; and Model 3 examined back muscle endurance. Interaction terms were calculated using mean-centered variables to reduce multicollinearity. Analysis was performed using SPSS version 20.0, with statistical significance set at *p* < 0.05. In addition, the PROCESS procedure was used to conduct simple slope analyses when interaction effects were significant.

## 3. Results

A total of 321 valid questionnaires were collected from a manufacturing factory. Among the participants, 87.2% were males and 12.8% were females, with an average age of 37.8 years (SD = 6.2). Most participants were married (67.3%), held a college or university degree (78.2%), reported an ordinary economic status (90.6%), and did not engage in shift work (77.6%). Regarding BMI, 1.6% were underweight, 36.1% had ideal weight, 32.7% were overweight, and 29.6% were categorized as obese. Table 1 also summarizes the distribution of demographic characteristics and the means of burnout and well-being across subgroups. Burnout differed significantly by marital status (*t* = −2.612, *p* < 0.01) and economic status (*F* = 4.331, *p* < 0.05). Well-being differed significantly by marital status (*F* = 3.534, *p* < 0.001) and economic status (*F* = 5.971, *p* < 0.01). No significant differences in burnout or well-being were observed across gender, educational level, BMI, or shift work.

Table 2 shows the descriptive statistics of the research variables. The average weekly work hours were 44.0 (*SD* = 4.2). Job control and psychological job demands had mean scores of 64.2 (*SD* = 9.0) and 31.4 (*SD* = 4.6), respectively. The mean scores for muscle fitness components were 3.63 (*SD* = 0.98) for grip strength, 3.95 (*SD* = 0.90) for abdominal muscle endurance, and 2.97 (*SD* = 0.96) for back muscle endurance. The mean levels of burnout and well-being were 41.0 (*SD* = 14.8) and 52.4 (*SD* = 19.7), respectively.

Table 3 displays the correlations among the research variables. Grip strength was significantly correlated with age (*r* = 0.149, *p* < 0.01), and abdominal muscle endurance (*r* = 0.184, *p* < 0.01). Back muscle endurance was positively correlated with age (*r* = 0.220, *p* < 0.01), job control (*r* = 0.120, *p* < 0.05), and abdominal muscle endurance (*r* = 0.378, *p* < 0.01). Burnout was correlated with weekly work hours (*r* = 0.142, *p* < 0.05), negatively correlated with job control (*r* = −0.165, *p* < 0.01), and positively correlated with psychological job demands (*r* = 0.480, *p* < 0.01). Well-being was positively correlated with job control (*r* = 0.245, *p* < 0.01) and negatively correlated with psychological job demands (*r* = −0.332, *p* < 0.01), and with burnout (*r* = −0.532, *p* < 0.01).

**Figure 1:**
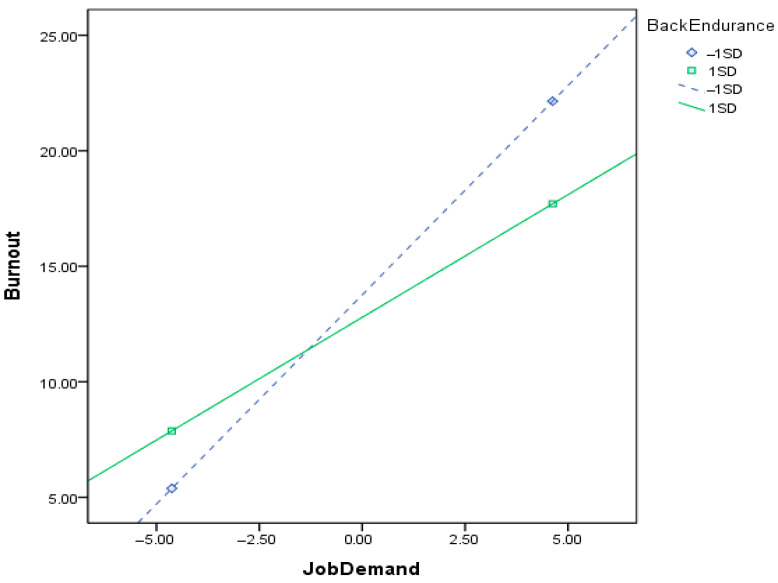
Simple slope analysis for relationships between job demand and burnout. Back endurance served as a moderator (the figure was made based on back endurance at one standard deviation above its mean, +1 SD, or one standard deviation below its mean, −1 SD). The figure indicated that when back endurance was at a lower level, a significant association between job demand and burnout occurred; when it was at a higher level, the association was decreasing.

Table 4 presents the results of multiple linear regression analyses predicting burnout. In Model 1, psychological job demands (β = 0.458, *p* < 0.001) and job control (β = −0.130, *p* < 0.05) were significantly associated with burnout, while neither grip strength nor its interaction terms with work stress were significant after controlling for covariates (*F* = 15.202, *p* < 0.001; adjusted *R*^2^ = 0.264). In Model 2, psychological job demands (β = 0.453, *p* < 0.001) and job control (β = −0.135, *p* < 0.01) remained significantly associated with burnout, whereas abdominal muscle endurance and its interaction terms were not significant (*F* = 14.698, *p* < 0.001; adjusted *R*^2^ = 0.262). In Model 3, back muscle endurance and its interactions with work stress were included. The interaction between job demand and back muscle endurance was significant (β = −0.121, *p* < 0.05), showing a significant interaction. The overall model remained significant (*F* = 15.837, *p* < 0.001; adjusted *R*^2^ = 0.278). All predictors in the regression models had very low variation inflation factor (VIF) ranging from 1.014 to 1.189 (<10), indicating no multicollinearity. Figure 1 illustrates the moderating role of back muscle endurance in the relationship between job demands and burnout. To further explore this interaction, a simple slope analysis was conducted. As shown in the figure (based on ±1 SD), the significantly positive association between job demands and burnout was more pronounced when back muscle endurance was lower. Statistical probing at ±2 SD further confirmed this pattern: at low levels of back muscle endurance (−2 SD), higher job demands were significantly associated with increased burnout. Conversely, at high levels of back muscle endurance (+2 SD), this association was attenuated and no longer reached statistical significance.

Table 5 summarizes the regression analyses predicting well-being. In all three models, higher job control (β = 0.190 to 0.208, *p* < 0.001) and lower job demands (β = −0.306 to −0.318, *p* < 0.001) were associated with better well-being after controlling for covariates. However, in contrast to the findings for burnout, neither the muscle fitness variables nor their respective interaction terms reached statistical significance (adjusted *R*^2^ ranged from 0.194 to 0.200; *p* < 0.001), indicating that muscle fitness did not moderate the relationship between work stress and well-being in this sample.

## 4. Discussion

The present study investigated the moderating role of specific muscle fitness components in the relationship between work stress and psychological outcomes among white-collar workers. The core finding was that back muscle endurance interacted with psychological job demands in relation to burnout levels, whereas no such statistical interactions were observed for grip strength and abdominal endurance. Furthermore, muscle fitness did not moderate the association between work stress and well-being. These results contribute to the occupational health literature by demonstrating that physical resilience against workplace strain is highly task-specific and context-dependent.

The robust association between psychosocial job characteristics and burnout/well-being reinforces the enduring validity of the JDC model [31,36]. Our results confirm that high job demands are associated with psychological strain, while job control serves as a vital resource for fostering well-being. In the context of the JDC framework, our findings suggest that when environmental job control is insufficient, individual-level physical resources may act as a critical secondary defense [42]. However, the absence of a direct correlation between muscle fitness and psychological outcomes aligns with the “stress-buffering hypothesis” [19,43]. This hypothesis suggests that physical fitness functions as a moderator that modifies the stress–strain relationship rather than serving as a direct antecedent of mental health. While cardiorespiratory fitness has been the primary focus of prior research [20,44], the current study extends this by showing that muscle fitness, as a distinct dimension, functions in a context-dependent manner [23].

A pivotal finding of this research is the selective moderating effect of back muscle endurance. Specifically, workers with superior back endurance showed greater resilience to the burnout-inducing effects of high job demands. This extends the JDC model [33] by demonstrating that physical endurance can function as a “functional control resource,” allowing workers to maintain postural stability and reduce the somatic cost of coping with high psychological demands [45]. In alignment with systematic reviews on upper extremity disorders, the interaction between physical load and psychosocial stressors is a critical determinant of musculoskeletal health [39]. Biomechanically, white-collar work is characterized by prolonged sedentary behavior and static loading, which impose significant strain on the spinal structures and the shoulder girdle [26,46]. Over time, sustained spinal flexion and muscle activation may contribute to “spinal creep” and localized muscular fatigue [47]. Individuals with insufficient back endurance may experience an earlier onset of postural instability and musculoskeletal discomfort [27,48]. From a psychosomatic perspective, we propose that this physical strain acts as a secondary “somatic stressor” [42]. When physical discomfort arises, it may deplete the individual’s limited self-regulatory resources, leaving them with less cognitive and emotional capacity to cope with psychological job demands, thereby exacerbating emotional exhaustion—the hallmark of burnout [3,29].

In contrast, the lack of moderation by grip strength and abdominal endurance underscores the principle of functional task-specificity. This aligns with the “Matching Principle” within JDC research, which posits that a resource must qualitatively match the type of demand to effectively buffer stress [49]. Grip strength is often regarded as a global indicator of vitality and aging [21,22], yet its relevance to the specific biomechanical demands of office work is limited. White-collar tasks primarily involve sustained trunk stabilization rather than forceful upper-limb exertion. Similarly, while abdominal strength contributes to core stability, the posterior back extensors are more continuously taxed during upright sitting to counteract gravitational flexion moments [24,28]. This implies that “global” fitness indices may be less effective for buffering occupational stress than “functional” fitness tailored to the specific biomechanical exposures of the job [23,24].

The failure of muscle fitness to moderate well-being further suggests that well-being may be predominantly shaped by psychosocial rather than physical resources within workplace contexts. According to the JDC model, while physical buffers can mitigate the “strain” (burnout) associated with high demands, the promotion of “active learning” and “well-being” typically requires environmental resources like job control and social support rather than just individual physical capacity [33]. This aligns with the notion that physical fitness may have divergent effects on different dimensions of well-being, such as positive flourishing versus the mitigation of ill-health [6,10]. Consistently, this distinction suggests that while physical conditioning can protect against “exhaustion” by reducing somatic strain, it may not inherently facilitate “flourishing” in the absence of favorable psychosocial conditions [10]. From a ‘Resource Matching’ perspective, muscle fitness provides a specific physical resource to counter biomechanical strain, yet well-being—as a higher-order cognitive-affective state—requires social and organizational resources like autonomy to flourish. Thus, physical capacity may function more as a ‘hygiene factor’ that prevents ill-health rather than a ‘motivator’ that linearly increases life satisfaction [50]. Addressing these distinctions is central to the current challenges in well-being science, emphasizing the need for multifaceted interventions [51].

Furthermore, a noteworthy observation in the present study is that the average well-being score of our participants was significantly lower than the reference scores reported for the European Union [2]. This discrepancy suggests that Taiwanese white-collar workers may face higher levels of psychological strain or have fewer available well-being resources compared to their European counterparts. The high-pressure work culture and long working hours prevalent in many Asian manufacturing contexts may contribute to this lower level of subjective well-being [52]. Specifically, the ‘diligence’ ethic and collectivist values in East Asian workplaces [53] may lead employees to internalize high job demands, potentially normalizing chronic exhaustion as a personal responsibility. From an occupational health management perspective, this suggests that interventions must move beyond individual physical training to address organizational cultures that may inadvertently reward overtime [54]. This cross-cultural well-being gap highlights substantial room for improvement in the psychological health of the Taiwanese white-collar workforce, requiring urgent attention from occupational health practitioners and organizational policy makers.

From a practical perspective, these findings suggest that burnout prevention programs should integrate ergonomic and physical components with traditional psychosocial interventions. Although the moderating effect size was modest, improving back muscle endurance through targeted training (e.g., core stabilization or posterior chain strengthening) represents a practically meaningful and cost-effective individual-level strategy to enhance resilience among employees in high-strain environments where organizational job redesign is difficult to implement immediately. Despite the rigorous design, this study has several limitations. First, the cross-sectional nature precludes causal assertions. Future research should employ longitudinal tracking or randomized controlled trials to confirm the extent to which muscle fitness training relates to reduction in burnout. Second, female representation in our sample (12.8%) was significantly lower than the national average in Taiwan’s manufacturing sector (37.8%) [55], potentially limiting generalizability to the broader female workforce. Furthermore, data collection from a single facility with prevalent long working hours and a ‘diligent’ East Asian work ethic may be associated with stress impacts differently than in Western contexts. Future studies should utilize stratified sampling across diverse industries to validate these findings. Third, the use of self-reported scales for burnout and job characteristics may introduce common-method or social desirability biases. Fourth, the absence of moderating effects on well-being may stem from measurement limitations; objective performance might not relate to global well-being as strongly as ‘perceived’ competence. Finally, although age and BMI were health proxies, we did not explicitly account for specific chronic diseases. Future investigations should incorporate medical screenings to further isolate the influence of muscle fitness on occupational stress buffering.

## 5. Conclusions

In conclusion, back muscle endurance functions as a critical moderator that buffers the adverse impact of high job demands on burnout among white-collar employees. This finding highlights a specific musculoskeletal pathway through which physical resilience is associated with a reduction in the translation of work stress into psychological exhaustion. However, the lack of association with well-being suggests that muscle fitness alone may be insufficient to promote positive indicators of mental health, which appear to remain primarily driven by psychosocial and organizational resources. Consequently, burnout prevention should not rely solely on individual physical conditioning. Integrative interventions that combine ergonomic adjustments, targeted spinal endurance training, and organizational strategies to enhance job autonomy and manage demands are recommended. Finally, given the cross-sectional nature of this evidence, longitudinal or experimental studies are essential to further clarify these relationships and evaluate the long-term efficacy of such multifaceted approaches in diverse occupational settings.

## Figures and Tables

**Table 1:** Comparing the intensity of burnout and well-being by the characteristics of the participants.

Variables			Burnout	Well-Being	
	n (%)	Mean (*SD*)	*t*/*F* (*ES*)	Mean (SD)	*t*/*F* (*ES*)
Gender			1.055 (*d* = 0.176)		−0.894 (*d* = 0.150)
Female	41 (12.8)	38.8 (12.6)		54.9 (19.3)	
Male	280 (87.2)	41.4 (15.1)		52.0 (19.7)	
Marital status			−2.612 ** (*d* = 0.311)		3.534 *** (*d* = 0.553)
Not married	105 (32.7)	44.1 (16.2)		46.9 (20.3)	
Married	216 (67.3)	39.6 (13.9)		55.0 (18.8)	
Educational level			0.377 (*η*^2^ = 0.008)		1.713 (*η*^2^ = 0.009)
Senior high	41 (12.8)	42. 9 (15.8)		47.1 (21.6)	
College/university	251 (78.2)	40.7 (14.8)		53.1 (19.4)	
Postgraduate	29 (9.0)	41.4 (13.6)		54.1 (18.8)	
Economic status			4.331 * (*η*^2^ = 0.016)		5.971 ** (*η*^2^ = 0.010)
Poor	15 (4.7)	51.7 (14.9)		38.1 (18.8)	
Ordinary	289 (90.6)	40.6 (14.9)		52.6 (19.6)	
Good	15 (4.7)	38.3 (8.7)		62.1 (15.0)	
BMI			0.956 (*η*^2^ = 0.010)		0.497 (*η*^2^ = 0.011)
Underweight	5 (1.6)	47.9 (14.2)		52.5 (19.4)	
Ideal weight	116 (36.1)	40.5 (16.1)		44.0 (14.1)	
Overweight	105 (32.7)	39.9 (13.5)		53.5 (18.4)	
Obesity	95 (29.6)	42.6 (14.7)		51.4 (21.6)	
Shift work			−0.433 (*d* = 0.058)		0.748 (*d* = 0.100)
Without	249 (77.6)	41.2 (14.1)		51.9 (19.0)	
With	72 (22.4)	40.4 (17.1)		53.9 (21.9)	

* *p* < 0.05; ** *p* < 0.01; *** *p* < 0.001. *ES* = Effect size. *d* = Cohen’s d. *η*^2^ = partial eta square.

**Table 2:** Means and standard deviations of research variables.

Variables (Possible Range)	Mean	*SD*
Age (20–65)	37.8	6.2
Weekly work hours (30–60)	44.0	4.2
Job control (24–96)	64.2	9.0
Job demand (12–48)	31.4	4.6
Grip strength (1–5)	3.63	0.98
Abdominal muscle endurance (1–5)	3.95	0.90
Back muscle endurance (1–5)	2.97	0.96
Burnout (0–100)	41.0	14.8
Well-being (0–100)	52.4	19.7

**Table 3:** The correlations of research variables.

Variables	1.	2	3	4	5	6	7	8
1. Age	--							
2. Weekly work hours	0.110 *	--						
3. Job control	0.072	0.230 **	--					
4. Job demand	−0.031	0.300 **	−0.026	--				
5. Grip strength	0.149 **	−0.003	−0.049	0.020	--			
6. Abdominal muscle endurance	−0.046	−00.062	−0.052	−0.017	0.184 **	--		
7. Back muscle endurance	0.220 **	0.023	0.120 *	−0.040	0.072	0.378 **	--	
8. Burnout	−0.081	0.142 *	−0.165 **	0.480 **	0.021	0.022	−0.064	--
9. Well-being	0.051	−0.088	0.245 **	−0.332 **	−0.045	−0.043	0.077	−0.532 **

* *p* < 0.05; ** *p* < 0.01.

**Table 4:** Summary of multiple linear regressions predicting the intensity of burnout.

	Model 1			Model 2			Model 3		
Variables	B	SE B	β	B	SE B	β	B	SE B	β
Constant	10.772	10.272		13.041	10.524		17.991	9.871	
Marital status	−4.201	1.552	−0.133 **	−4.393	1.537	−0.141 **	−4.225	1.531	−0.135 **
Economic status	−3.748	2.398	−0.078	−3.839	2.409	−0.080	−3.913	2.361	−0.082
Weekly work hours	0.164	0.187	0.046	0.147	0.187	0.042	0.133	0.185	0.038
Job control	−0.215	0.084	−0.130 *	−0.227	0.085	−0.138 **	−0.244	0.084	−0.148 **
Job demand	1.477	0.165	0.458 ***	1.439	0.166	0.453 ***	1.437	0.164	0.452 ***
Grip strength	0.234	0.736	0.015						
Job control × grip strength	−0.098	0.085	−0.057						
Job demand × grip strength	−0.042	0.154	−0.013						
Abdominal muscle endurance				0.446	0.806	0.027			
Job control × abdominal endurance				−0.013	0.086	−0.008			
Job demand × abdominal endurance				−0.159	0.169	−0.047			
Back muscle endurance							−0.518	0.760	−0.034
Job control × back endurance							0.111	0.077	0.071
Job demand × back endurance							−0.390	0.159	−0.121 *
Adjusted *R*^2^	0.264			0.262			0.278		
*F*	15.202 ***			14.698 ***			15.837 ***		

Notes: Β denotes unstandardized regression coefficient; SE denotes standard error; β denotes standardized regression coefficient. Not married was coded as a referent. * *p* < 0.05; ** *p* < 0.01; *** *p* < 0.001.

**Table 5:** Summary of multiple linear regressions predicting the intensity of well-being.

	Model 1			Model 2			Model 3		
Variables	B	SE B	β	B	SE B	β	B	SE B	β
Constant	50.087	12.374		48.584	12.773		41.540	11.997	
Marital status	7.119	2.145	0.170 **	7.359	2.137	0.178 **	6.929	2.152	0.167 **
Economic status	8.491	3.311	0.133 *	7.936	3.344	0.124 *	7.832	3.312	0.123 *
Job control	0.415	0.113	0.190 ***	0.432	0.115	0.197 ***	0.456	0.115	0.208 ***
Job demand	−1.357	0.216	−0.318 ***	−1.305	0.219	−0.309 ***	−1.295	0.218	−0.306 ***
Grip Strength	−1.027	1.017	−0.051						
Job control × grip strength	0.000	0.117	0.000						
Job demand × grip strength	0.244	0.213	0.058						
Abdominal muscle endurance				−1.045	1.119	−0.048			
Job control × abdominal endurance				0.030	0.120	0.013			
Job demand × abdominal endurance				0.258	0.235	0.058			
Back muscle endurance							0.532	1.068	0.026
Job control × back endurance							0.006	0.109	0.003
Job demand × back endurance							0.349	0.223	0.082
Adjusted *R*^2^	0.200			0.194			0.196		
*F*	12.317 ***			11.612 ***			11.726 ***		

Notes: Β denotes unstandardized regression coefficient; SE denotes standard error; β denotes standardized regression coefficient. Not married was coded as a referent. * *p* < 0.05; ** *p* < 0.01; *** *p* < 0.001.

## Data Availability

The research data will be available upon request to the corresponding author. The data are not publicly available due to privacy or ethical restrictions.
